# The 2023 Arnold Berliner Award for research on spider photoreceptors

**DOI:** 10.1007/s00114-023-01870-9

**Published:** 2023-08-02

**Authors:** Matthias Waltert

**Affiliations:** Department of Conservation Biology, University of Göttingen, Bürgerstrasse 50, 37073 Göttingen, Germany

**Fig. 1:**
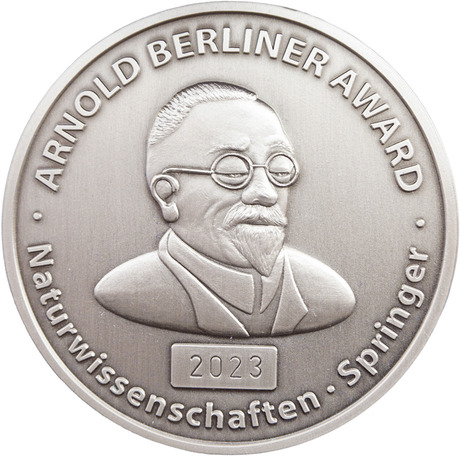
The Arnold Berliner Award medal

As every year, *The Science of Nature* grants the Arnold Berliner Award to the lead author of an article which represents excellent, original, and—especially—interdisciplinary research. As such, the winning articles clearly reflect the vision of Arnold Berliner (Autrum 1988; Thatje 2018). Springer sponsors the award which consists of four parts: the Arnold Berliner Award medal (Fig. 1), a 2-year subscription to the journal’s electronic edition, a 500-Euro voucher for Springer ebooks, and a cash prize of 250 Euro.

**Fig. 2:**
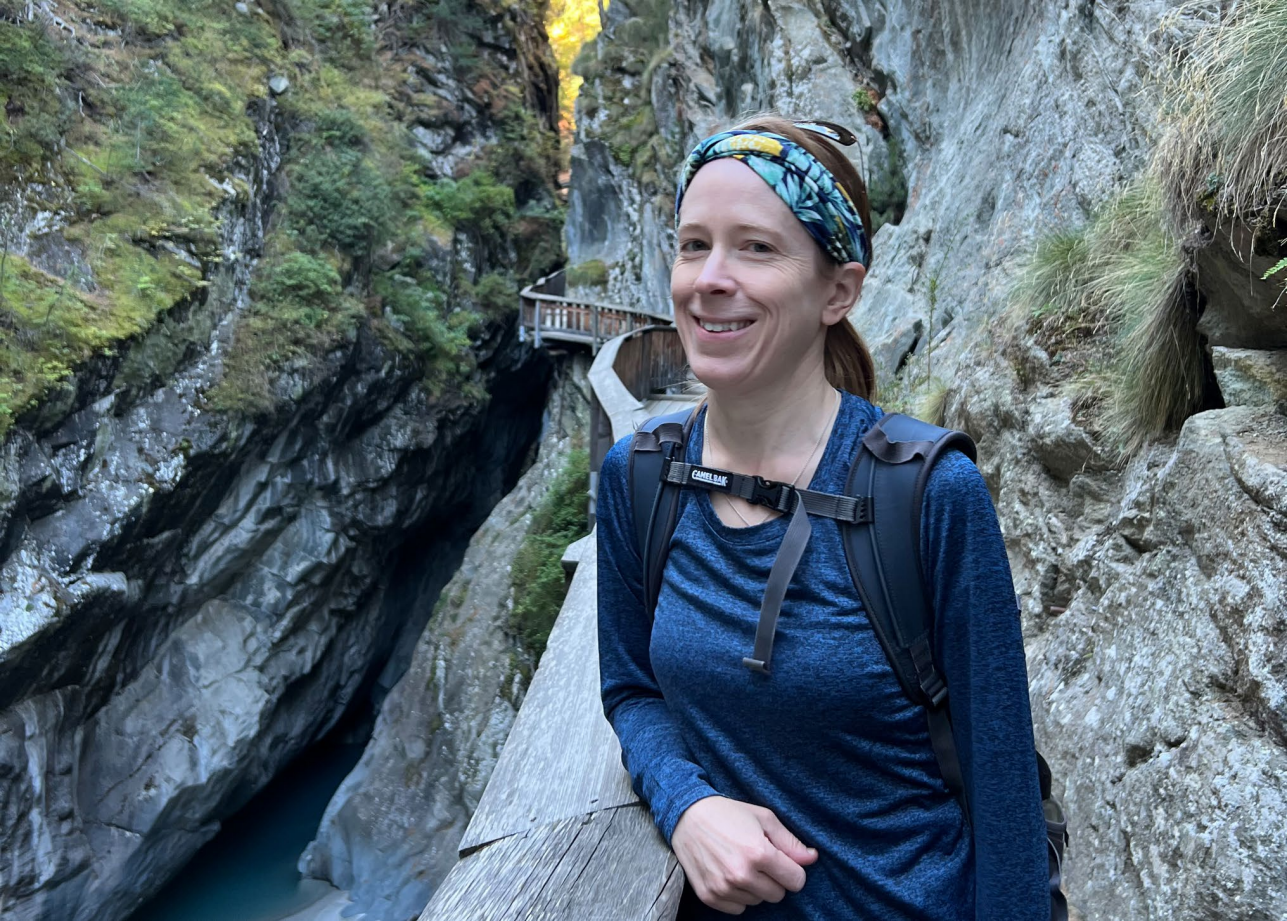
Cynthia Tedore

I am very proud that, this year, the Editorial Board of our journal has decided to award Cynthia Tedore (Fig. 2) for the article “The jumping spider Saitis barbipes lacks a red photoreceptor to see its own sexually dimorphic red coloration” (Glenszczyk et al. 2022). With their research, the authors suggest that jumping spiders cannot see the strikingly (to the human eye) red colored patches on males that were previously thought to function as signals in mate choice by females. The often, to us, striking coloration of animals leads to equally strong hypotheses about these colors’ significance. However, as the study finds, sometimes striking colors have different or even no meanings. The red coloration of the studied jumping spider’s is not visible to its female, refuting the idea that this color per se has a function in the sexual context. In contrast, some to the human eye achromatic color patches absorb or reflect UV and create strong contrasts which are visible to the spider.

On behalf of the Editorial Board, I congratulate Cynthia Tedore, the two co-first authors Mateusz Glenszyk and David Outomuro, and the rest of the team on the award.
